# Auditory-Induced Negative Emotions Increase Recognition Accuracy for Visual Scenes Under Conditions of High Visual Interference

**DOI:** 10.3389/fpsyg.2018.02374

**Published:** 2018-11-27

**Authors:** Oliver Baumann

**Affiliations:** ^1^Queensland Brain Institute, The University of Queensland, Brisbane, QLD, Australia; ^2^Interdisciplinary Centre for the Artificial Mind, School of Psychology, Bond University, Gold Coast, QLD, Australia

**Keywords:** memory, valence – arousal, emotion, scenes, memory accuracy

## Abstract

The effect of emotion on memory is powerful and complex. While there seems to be agreement that emotional arousal generally increases the likelihood that events are remembered, it is somewhat disputed whether also the valence of emotions influences memory. Specifically, several experiments by Kensinger and colleagues have provided evidence for the hypotheses that negative valanced emotions facilitate the encoding of perceptual details. On the other hand, Mather and colleagues have suggested that these results could be explained by confounding relationships of valence and arousal, i.e., that items that generate negative emotions are typically also more arousing. In this study, we provide a conceptual replication of Kensinger’s findings. We employed a novel experimental design, in which the effects of standardized emotional arousing sounds on recognition accuracy for neutral visual scenes was measured. We indirectly manipulated the amount of visual detail that was encoded, by requiring participants to memorize either single exemplars (low interference) or multiple exemplars (high interference) of visual scene categories. With increasing visual overlap in the high interference condition, participants were required to encode a high degree of visual detail to successfully remember the exemplars. The results obtained from 60 healthy human participants confirmed Kensinger’s hypothesis by showing that under conditions of high visual interference, negative valanced emotions led to higher levels of recognition accuracy compared to neutral and positive emotions. Furthermore, based on the normative arousal ratings of the stimulus set, our results suggest that the differential recognition effect cannot be explained by differing levels of arousal.

## Introduction

Emotions can be categorized based on two dimensions (Lang et al., 1990; Lang, 1995): arousal (i.e., calming vs. exiting) and valence (positive vs. negative). While it has been shown that elevated arousal during encoding increases the chance of remembering a particular stimulus (Kensinger et al., 2002), valence seems to be an important moderator for the amount of details that are remembered (Kensinger, 2009a,b). Specifically, while positive valence seems to have little effect, negative valence appears to significantly increase the amount of details that are remembered. For example, Kensinger et al. (2007) have shown that participants are more accurate in remembering visual details of negative valanced items (e.g., “a green and black snake with yellow eyes”) compared to positive items (e.g., “a cake”). Obviously, there could be visual confounds that might lead to more detailed memory for negative compared to positive stimuli. It has been shown, however, that even if visual confounds are controlled for, the effect of valence on memory accuracy prevails. One prominent example is the study by Kensinger and Schacter (2006), which measured the amount of details Red Sox fans and Yankee fans could report from a baseball game and found that supporters of the losing team had more detailed memories. In this instance, the visual information was matched between the groups, but what differed was the either positive or negative appraisal of the event that led to differences in memory accuracy. Furthermore, neuroimaging evidence (Kensinger and Schacter, 2008; Mickley and Kensinger, 2008) showed that encoding of negative items that were later remembered led to increased activity in sensory brain areas, but this was not the case for neutral or positive emotions, which indicates that negative valence facilitated the encoding of perceptual details. It is, however, important to note that Mather and Sutherland (2009) argue that negative stimuli and events are typically confounded with higher levels of arousal and that arousal appears to be the better predictor of memory accuracy than valence. More specifically, Mather and Sutherland (2011) propose that at high levels of arousal, positive, negative and even neutral stimuli would have similar effects on how information is encoded (Sutherland and Mather, 2018).

Previous research has operationalized the accuracy of memory by measuring the number of details that participants could report. The goal of the current study was to provide a conceptual replication of the effect of emotional valence on memory by using a novel method to quantify the accuracy of memories. Konkle et al. (2010) asked participants to encode thousands of scene images. By varying the number of exemplars presented per scene category and testing memory using exemplar-level foils they observed a 2% decrease in memory performance for each doubling of the number of studied scene exemplars per category. In contrast, performance was found to be unaffected by the addition of further single image categories. We recently corroborated these findings (Baumann et al., 2018), by showing that participants’ recognition accuracy is generally less accurate for scenes of which 10 category members (high interference) have been presented, compared to scenes of which just a single category member (low interference) has been presented during encoding. For participants to be able to remember the images in the high interference condition they cannot focus on the gist or semantic category but must encode visual details to be successful. Varying levels performance in the high vs. low interference conditions can be therefore used as an indirect marker of the amount of detail encoded.

The goal of the present study was to use the methodology introduced by Konkle et al. (2010) to assess whether negative emotional valence leads to an increase in the amount of details remembered compared to positive and neutral valence. To avoid any potential confounding effects between the visual aspects of the stimuli and the type and degree of emotion they elicit we employed auditory stimuli to induce emotional states and measured the memory accuracy for neutral scene images.

## Methods

### Participants

The study was aimed to include 60 participants. For the study, 72 adults from the University of Queensland gave informed consent and were compensated for their participation with course credit. Seven participants failed to show up for the recognition session and five did not meet the minimum *a priori* performance criterion of 70% accuracy in the low interference condition (averaged over all three emotions). 60 participants were included in the final analysis [M_age_ = 24.5 years (*SD* = 5.1), 18 male].

### Stimuli

Stimuli were 288 scene images (50% indoor and 50% outdoor) collected using Google image search. We manipulated interference by varying the number of images in a semantic category, following Konkle et al. (2010). During encoding, low interference conditions were comprised of 72 images from 72 semantic categories, and high interference conditions were comprised of 72 images from only 9 semantic categories (8 images per category). Three counterbalancing schemes were employed, so that over all participants there was an equal number of indoor and outdoor scenes for the high and low interference condition. Every image was paired with a similar ‘foil’ picture during the recognition test. Target-foil pairings were selected to be highly similar, based on features such as spatial distribution, texture, color, image quality, and object categories in the scene.

To induce positive, negative and neutral affective states, we employed sounds from the International Affective Digital Sounds (IADS; Bradley and Lang, 2007) stimulus set. These stimuli include normative valence and arousal ratings on a 9-point scale from at least 100 participants. We selected eight sounds that were of positive valence and high arousal [i.e., mean valence = 7.27 (*SD* = 0.44); mean arousal = 7.11 (*SD* = 0.25)]; eight sounds that were of negative valence and high arousal [mean valence = 1.78 (*SD* = 0.195); mean arousal = 7.80 (*SD* = 0.27)], and eight sounds that were of neutral valence and low arousal [mean valence = 6 (*SD* = 0.98), mean arousal = 3.49 (*SD* = 0.32)]. The sounds all had a duration of 6 s and were played at a comfortable sound pressure level. The visual and auditory stimuli were presented using Presentation stimulus delivery software, version 16.2 (*Neurobehavioral Systems*). Using three counterbalancing schemes we ensured that over all participants each scene image was presented equally often with positive, negative and neutral sounds.

### Procedure

**FIGURE 1:**
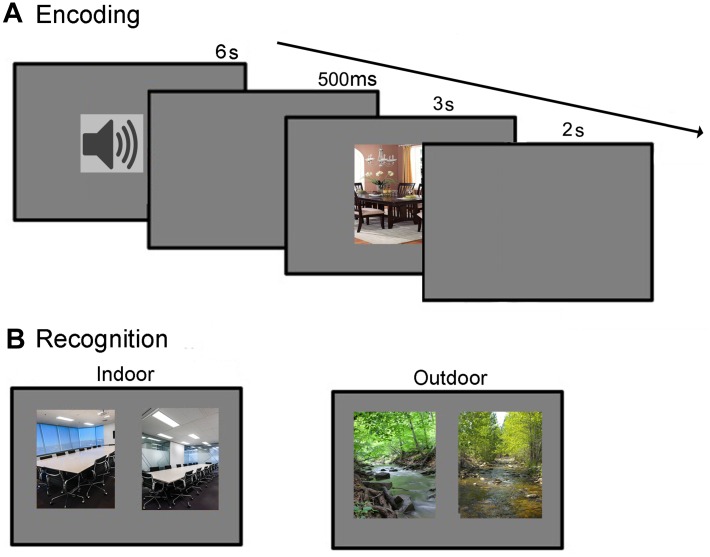
**(A,B)** Encoding and recognition procedures.

During encoding, each trial started with the presentation of an affective sound (6 s duration), followed by a 500 ms silent interval (see Figure 1A). After the interval, a scene image was presented for 3 s. Participants were asked to only attend to the scene image and were told that they would be subsequently asked to recognize them. One day after the encoding session, the participants were undergoing a recognition test. This test phase consisted of 144 2AFC trials. In each trial, two images from the same scene category were presented horizontally next to each other–one was a previously studied target image, and the other a distractor image that participants had not seen before (see Figure 1B). Participants were instructed to indicate which of the two scenes they had previously studied. No feedback was provided. In half of the trials the target image was on the left side of the screen and the distractor on the right, and vice versa for the other half of the trials. The presentation order of the stimulus pairs was randomized for each participant. Participants proceeded at their own pace and were told to emphasize accuracy, not speed.

## Results

**FIGURE 2:**
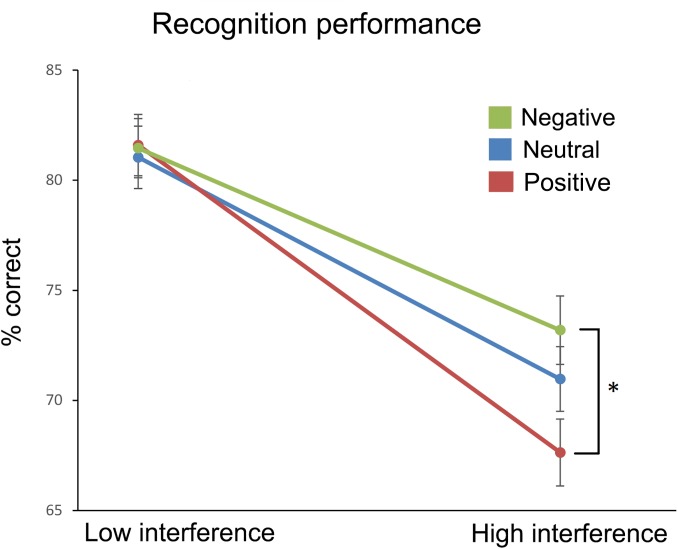
Percentages of correct recognition ( ± 1 SE) for the three affective conditions that were presented under low and high levels of visual interference. ^∗^Statistically significant difference (*p* < 0.025, two-tailed paired *t*-test).

Recognition performance is plotted in Figure 2. To test for effects of emotion (positive, negative, neutral) and interference (high, low), we conducted a 3 × 2 repeated measures ANOVA. We observed a significant main effect of interference, *F*(1,59) = 134.770, *p* < 0.001, $\eta_{p}^{2}$ = 0.696, but only a trend for emotion, *F*(2,118) = 3.006, *p* = 0.053, $\eta_{p}^{2}$ = 0.048. Importantly, their interaction was significant, *F*(2,118) = 3.362, *p* = 0.038, $\eta_{p}^{2}$ = 0.054. Two planned follow-up paired *t*-tests (using Bonferroni-adjusted alpha levels of 0.025 per test) showed that recognition accuracy was significantly lower *t*(59) = 3.178, *p* = 0.002, *d* = 0.41, for positive than negative emotion under high interference (67.6% vs. 73.1%, SEMs = 1.5%), but there was no difference *t*(59) = 0.125, *p* = 0.901, *d* = 0.01, under low interference (81.6% vs. 81.5%, SEMs 1.4 and 1.3%, respectively).

## Discussion

The goal of this study was to conceptually replicate findings by Kensinger and colleagues (c.f. Kensinger, 2009a,b), which suggested that negative emotions engage sensory processes and focus attention on intrinsic details, while positive emotions lead to the encoding of the gist of stimuli, while details are forgotten. Our experimental design included several features to assess the generalizability of these earlier findings and to investigate potential criticisms that have been voiced in the past. Firstly, we used a novel approach to indirectly assess the amount of detail that was remembered under positive and negative emotional valence. Secondly, we used auditory affective stimuli to induce emotions and tested memory for neutral visual stimuli, avoiding potential visual confounds that could occur if the visual stimulus material itself is affective laden.

We found that negative induced affect led to higher accuracy than positive induced affect for images that required the encoding of a high level of visual detail (high interference). In the mnemonic condition which required a less detailed representation (low interference) there was no difference between the positive and negative emotion conditions. Importantly, memory performance in the positive emotion condition was even worse than in the low-arousal control condition, which indicates that the better performance in the negative compared to positive emotion condition cannot be explained by differences in arousal. On the other hand, the comparison to the control condition, also suggest that the effect of valence on memory accuracy seems to not only be driven by a facilitation associated with negative valence, but also by an impairment associated with positive valence. This effect can be explained by previous studies that have shown that positive valence leads to a focus on gist rather than detail (e.g., Rowe et al., 2007; Yegiyan and Yonelinas, 2011).

In summary, using a novel experimental design we provided support for Kensinger’s (2009a,b) hypothesis that, independent of arousal, negative affect facilitates the encoding of sensory details. A limitation of the current study is that we relied on normative ratings of arousal and valence rather than monitoring actual physiological arousal in the participants, which could reveal additional information, such as potentially differential rates of habituation to positive and negative stimuli. Finally, the found effects are significant, but relatively modest in effect size. We believe that this could be due to the difficulty of inducing strong emotions with standardized experimental stimulus material, which might also explain why other studies did not find this effect (e.g., Sutherland and Mather, 2018). It is also important to note that Mather and colleagues have suggested that emotional arousal should have an enhancing effect on recognition performance only when stimuli are goal relevant (Sakaki et al., 2014), which was not manipulated in our study. Future neuroimaging studies could investigate the effect of emotional valence on memory encoding of highly similar stimulus material to provide corroborative neural evidence.

## Data Availability Statement

The data and program code is available at Open Science Framework (doi: 10.17605/OSF.IO/W3NJG).

## Ethics Statement

All subjects gave a written informed consent in accordance with the Declaration of Helsinki. The study was approved by the Human Research Ethics Committee of The University of Queensland.

## Author Contributions

OB was responsible for study design, data acquisition, data analysis, and manuscript writing.

## Conflict of Interest Statement

The author declares that the research was conducted in the absence of any commercial or financial relationships that could be construed as a potential conflict of interest.
